# Editorial: Ozone as a Driver of Lung Inflammation and Innate Immunity and as a Model for Lung Disease

**DOI:** 10.3389/fimmu.2021.714161

**Published:** 2021-06-30

**Authors:** Kian Fan Chung, Dieudonnée Togbe, Bernhard Ryffel

**Affiliations:** ^1^ Experimental Studies, National Heart & Lung Institute, Imperial College, London, United Kingdom; ^2^ Laboratory of Experimental and Molecular Immunology and Neurogenetics, UMR 7355 CNRS-University of Orleans, Orléans, France

**Keywords:** ozone, air pollution, chronic obstructive pulmonary disease, emphysema, lung inflammation, corticosteroid insensitivity, oxidative stress

The articles in this series will remind readers of the increasing importance of ozone as an important component of air pollution that contributes to mortality and disease progression (1–3).

Ground level ozone is created by chemical reactions in the presence of sunlight between oxides of nitrogen (NOx) and volatile organic compounds (VOC) that are both emitted as pollutants from cars, power plants, industrial boilers, refineries, chemical plants, and other sources. Levels of ozone are most likely to reach unhealthy levels on hot sunny days in urban areas, although high levels may also be observed during winter months. Ozone can also be transported long distances by wind into non-urban areas.

The effects of ozone on health are relatively well-known. Exposure to ozone can cause difficulty to breathe, shortness of breath and discomfort on breathing, cough and sore-throat presumably due to an inflammation and damage to the upper and lower airways. Ozone exposure can aggravate lung conditions such as asthma and COPD, causing acute deterioration of these conditions that necessitate emergency treatments (4, 5). As such, ozone exposure is likely to cause increased school absences, days off work, medication use, visits to doctors and emergency rooms, and hospital admissions.

Ozone is highly reactive, eliciting rapid and dose-dependent disruption of the respiratory barrier. It impacts many cell types in the lung and activates specific signaling cascades, eliciting responses including cellular damage, enhanced apoptosis, cytokine production, recruitment of inflammatory cells, and subsequent tissue repair.

Oxidative stress is a conserved mechanism that contributes to numerous environmental lung injuries. Ozone, as a principal mediator of oxidative stress in both the intracellular and extracellular compartments, has become a clinically-relevant model to understand the mechanisms underlying biological responses to oxidative stress (6, 7). Oxidation products are either directly toxic and can cause injury to lung tissue or they can function as exogenous ligands *via* binding to cell surface receptors and thereby triggering intracellular inflammatory and/or apoptotic signaling pathways (8). Thus, ozone‐induced oxidant stress modifies several known cell‐signaling mechanisms: activation of innate immune signaling pathways, upregulation of antioxidant genes, and enhanced release of damage‐associated molecular pattern molecules (DAMPs) (9, 10). Oxidative stress also decreases the clearance of pathogens by impairing antimicrobial function of effector cells including suppressing alveolar macrophage phagocytosis, enhancing macrophage and neutrophil apoptosis, and increasing the susceptibility of epithelial cells to influenza infection.

This Research Topic collection on ozone extends our knowledge on ozone of the recent decade. We summarize the contents of each of these 9 articles.

Dr. J. J. Zhang et al. starts this collection stating that ozone is responsible for hundreds of thousands of premature deaths and tens of millions of asthma-related emergency visits annually. To combat ozone pollution globally, an urgent reduction in fossil fuel consumption is required to cut NO_x_ and VOCs and greenhouse gas emissions. Preventive and therapeutic strategies are to alleviate the detrimental effects of ozone especially for susceptible individuals need to be further explored. The efficacy of antioxidants, and the major pathogenic pathways need to be investigated to identify molecular target.

Dr. C. Michaudel et al. revisits respiratory epithelial damage and inflammation in mice and extended previous knowledge showing that a single ozone (1h, 1ppm) causes within hours epithelial cell death with IL-1α and IL-33 expression followed by neutrophilic inflammation and repeated exposure over 6 weeks in mice induced chronic respiratory pathology with chronic inflammation and emphysema dependent on aryl hydrocarbon and IL-17/IL-22 expression.

Dr. M. Sokolowska et al. add new insights on the respiratory barrier disruption of epithelial tight junctions and cell death followed by ROS activation, airway hyperreactivity (AHR), myeloid cell recruitment and remodeling. High ROS levels activate a novel PGAM5 phosphatase dependent cell-death pathway, called oxeiptosis. Chronic ozone exposure leads to progressive and irreversible loss of alveolar epithelial cells and alveoli resulting in reduced gas exchange space typical of emphysema.

Dr. Wiegman et al. review oxidative stress in the lung upon acute exposure and chronic ozone exposure the activation of oxidative pathways causes AHR, cell death tissue remodeling, emphysema and chronic inflammation mimicking cigarette smoke induced COPD. There is derangement of the integrity of cell membranes and organelles of the respiratory epithelial cells causing a stress response with the release of mitochondrial reactive oxygen species (ROS), DNA, and proteases. Mitochondrial ROS and DNA activate NLRP3 inflammasome and the DNA sensors cGAS and STING accelerating inflammation enhancing alveolar septa destruction, remodeling, and fibrosis. Inhibitors of mitochondrial ROS, NLRP3 inflammasome, DNA sensor and cell death pathways may represent novel therapeutic targets.

Dr. Flayer et al. report on the effect of ozone inhalation impairing glucocorticoid responsiveness in a mouse model of allergic asthma induced by sensitization and challenge with Aspergillus fumigatus. Ozone exposure counteracted the effects of budesonide on airway inflammation, airway hyperreactivity, and surfactant protein D (SP-D) production. Asthmatics who are exposed to high ambient ozone levels may become less responsive to glucocorticoid treatment particularly during acute exacerbations of asthma.

Dr. Mumby et al. report on acute ozone exposure in man resulting in sputum neutrophilia, respiratory irritation and may be associated with systemic inflammation and chronic exposure amplifies these effects. In asthmatic subjects, ozone induces a greater number of genes in bronchoalveolar macrophages than healthy responders with up-regulation of inflammatory and immune pathways and the enhanced expression of repair programs. Models of ozone exposure recapitulate the inflammatory effects seen in humans and enable the elucidation of key transcriptional pathways that drive ozone effects on airways.

Dr. Enweasor et al. review oxidative stress in neutrophil recruitment and their resistance to glucocorticosteroids and innate lymphoid cells (ILC). Furthermore, a role of ILC type 2 cells was reported in the ozone inflammatory response, but also of ILC type 1 such as NK cells as well as NKT cells. Anti-CD1d mAb administration blocked NKT cell activation and ozone-induced AHR asthma. NKT cells mediate a unifying pathogenic mechanism for several distinct forms of asthma.

Dr. Shore investigates the metabolic effects of ozone on glucose intolerance and hyperlipidemia, characteristics of the metabolic syndrome. Further, the role of stress hormones and exacerbation by obesity and diabetes. The intestinal microbiome is critical in the regulation of metabolism is well known and a link of the gut microbiome and pulmonary inflammation in response to ozone has been uncovered including heightened AHR

Dr. Noutsios et al. review surfactant protein A (SP-A) in the airway and the interaction with alveolar macrophages (AM), the guardian cells of innate immunity in the lungs. SP-A regulates many of its functions under basal condition and in response to infection and oxidative stress. They investigated the effect of hSP-A variants on the AM gene expression profile in response to Klebsiella pneumoniae infection and differences in AM gene expression of TP53, TNF, and cell cycle.

## Author Contributions

All authors contributed to the article and approved the submitted version.

## Conflict of Interest

The authors declare that the research was conducted in the absence of any commercial or financial relationships that could be construed as a potential conflict of interest.
